# Trisomy 8: a common finding in mouse embryonic stem (ES) cell lines

**DOI:** 10.1186/1755-8166-6-3

**Published:** 2013-01-16

**Authors:** Young Mi Kim, Ji-Yun Lee, Lijun Xia, John J Mulvihill, Shibo Li

**Affiliations:** 1 Department of Pediatrics, The University of Oklahoma Health Sciences Center, Oklahoma City, OK 73104, USA; 2 Cardiovascular Biology Research Program, Oklahoma Medical Research Foundation, Oklahoma City, OK, 73104, USA; 3 Department of Pathology, Korea University, Seoul, 136-705, South Korea

**Keywords:** Mouse ES cells, Chromosomal aberrations, FISH, Mosaicism

## Abstract

**Background:**

Obtaining a germ cell line is one of the most important steps in developing a transgenic or knockout mouse with a targeted mutated gene of interest. A common problem with this technology is that embryonic stem (ES) cells often lack, or are extremely inefficient at, germ line transmission.

**Results:**

To determine whether chromosomal anomalies are correlated with inefficient ES cell germ line transmission, we examined 97 constructed ES cell lines using conventional cytogenetic analysis, and fluorescence *in situ* hybridization (FISH). Chromosomal abnormalities occurred in 44 (45%) out of the 97 specimens analyzed: 31 specimens had trisomy 8 or mosaic trisomy 8, eight specimens had partial trisomy 8 resulting from unbalanced translocations, and five specimens had other chromosomal anomalies.

**Conclusions:**

Our data suggest that chromosomal analysis is an important tool for improving the yield and quality of gene targeting experiments.

## Background

Although the whole human genome has now been sequenced, determining the function of each gene in the human body remains a challenge. A practical and frequent approach to studying human gene function is to use mouse models, accomplished by direct mutagenesis through targeting mouse embryonic stem (ES) cells. Established mouse ES cell lines have the ability to maintain unlimited proliferation *in vitro* and differentiate into a variety of cell lineages including germ cells [1]. The ability to obtain a germ line is one of the most important steps in developing a transgenic or knockout mouse with a specific mutated gene. However, a common problem with this valuable technique is that the ES cells often lack or have a low efficiency of germ line transmission. Thus, understanding what affects the efficiency of germ line transmission is crucial to developing transgenic and knockout mice.

Many factors determine the efficiency of germ line transmission [2]. Chromosome make-up clearly affects both somatic cell chimerism and germ line transmission. For example, ES cells with trisomy 8 are significantly less efficient at achieving other germ line transmission than cells with normal karyotypes [3]. Aneuploid ES cells have very low germ line transmission [4]. Chimeric mice obtained from chromosomally abnormal ES cells often have phenotypic abnormalities beyond those of pretargeted gene [5]. This abnormal genotype makes correlating the genotype and phenotype in chimeric mice extremely difficult, if not impossible.

It is not clear why structural and numerical chromosome abnormalities are found in ES cell lines subjected to extended culture *in vitro*. One hypothesis is that the changes confer a proliferative benefit to those cells (favorable selection). Cell aging is another possible explanation. The frequency of chromosomal anomalies increases with passage of cells in culture [4,6]; for example, a *de novo* Robertsonian translocation between homologous chromosomes 11 was spontaneously induced [7].

To qualify this troublesome phenomenon, we analyzed the chromosome of 97 mouse ES cell lines using conventional cytogenetic technique and fluorescence *in situ* hybridization (FISH).

## Results

Of the 97 ES cell lines examined, 44 (45%) had either numerical or structural changes or a combination of both (Table 1). Among these abnormal ES cell lines, 31 had numerical changes, seven cell lines had structural changes and six cell lines had both numerical and structural changes.

**Table 1:** Summary of cytogenetic findings in all 97 mouse ES cell lines

**Cytogenetic findings**	**Number of cell lines**	**%**
Normal karyotype	53	55
Numerical changes (Table 2)	31	32
Structural changes (Table 3)	7	7
Both numerical and structural changes (Table 4)	6	6
**Total**	**97**	**100**

**Figure 1:**
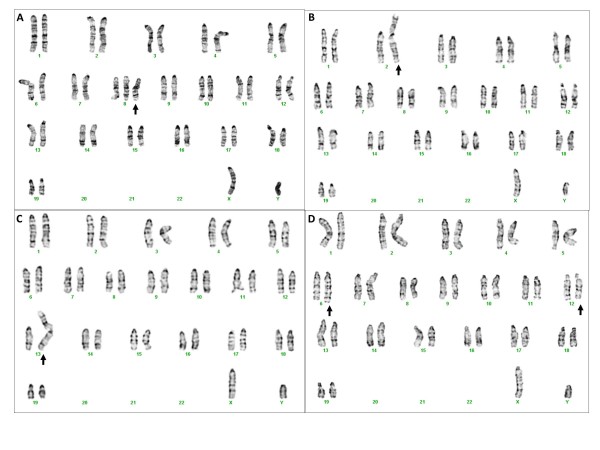
**Conventional G-banded karyotypes with numerical and structural anomalies.** (**A**): 41,XY,+8. (**B**): 40,XY,der(2)t(2;8)(A1;A1). (**C**): 40,XY,der(13)t(8;13)(A1;A1). (**D**) 40,XY,t(6;12)(F3;D3). Arrows show the abnormal chromosomes.

In 31 cell lines with numerical changes, 16 (52%) were pure trisomy 8 (41,XY,+8) (Table 2, Figure 1A), six had mosaic trisomy 8 (41,XY,+8/40,XY) and two had double trisomies of chromosomes 8 and 11 (42,XY,+8,+11). The remaining seven cell lines had either mosaic or pure trisomies of chromosome 8 (6/7), chromosome 11 (4/7) or chromosome Y (3/7). One cell line had a loss of the Y chromosome.

**Table 2:** Summary of numerical anomalies in mouse ES cell lines

**Numerical anomalies**	**Number of cell lines**
43,XY,+Y,+8,+11	1
41,XY,+Y/42,XY,+Y,+8/40,XY	1
41,XY,+Y/41,XY,+8/42,XY,+3,+7	1
40,X,-Y,+8/40,X,-Y,+11/41,X,-Y,+6,+8/41,X,-Y, +6,+11/42,XY,+6,+8/42,XY,+6, +11/40,XY	1
41,XY,+8	16
41,XY,+8/40,XY	6
42,XY,+8,+11	2
42,XY,+8,+15	1
41,XY,+11	1
44,XY,+1,+6,+8,+11	1
**Total**	**31**

**Figure 2:**
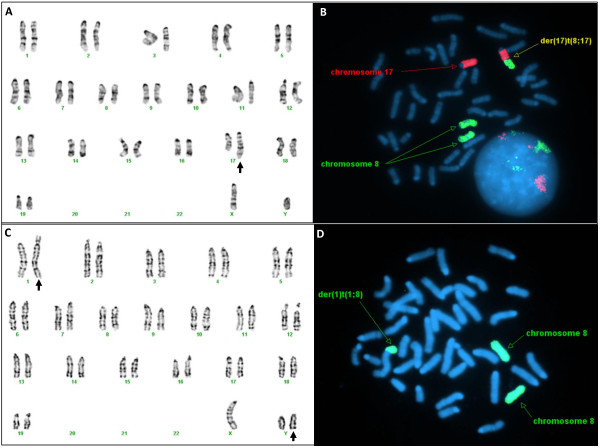
**Karyotype and confirmatory FISH analysis for unbalanced translocation.** Unbalanced translocation between chromosome 8 and chromosome 17 [40,XY,der(17)t(8;17)(B2;E2)], that is indicated by arrow (**A**). FISH using WCP 8 (green) and WCP 17 (red) confirmed two green signals for normal chromosome 8, one normal red signal for normal chromosome 17 and one derivative chromosome 17 due to the unbalanced translocation between 8 and 17, which is painted by partial red and green (**B**). Unbalanced translocation between chromosome 1 and chromosome 8 [41,XY,+Y,der(1)t(1;8)(A1;D1), that is indicated by arrow (**C**). FISH using WCP 8 (green) confirmed two green signals for normal chromosome 8 and one derivative chromosome 1 due to the unbalanced translocation between 1 and 8, which is painted by green partially (**D**).s

Seven of the 97 cell lines (7%) had structural abnormalities (Table 3). Two of these seven cell lines had Robertsonian translocations between chromosomes 2 and 8 [40,XY,der(2)t(2;8)(A1;A1)] (Figure 1B) and chromosomes 8 and 13 [40,XY,der(13)t(8;13)(A1;A1)] (Figure 1C). One cell line had an apparently balanced translocation between chromosomes 6 and 12 [40,XY,t(6;12)(F3;D3)] (Figure 1D). Two cell lines had a derivative chromosome 17 due to an unbalanced translocation between chromosomes 8 and 17 [40,XY,der(17)t(8;17)(B2;E2)] (Figure 2A), which was also confirmed by FISH (Figure 2B). One cell line had a mosaic interstitial deletion of the long arm of chromosome 6 at the breakpoints of B1 and C3 [40,XY,del(6)(B1C3)/40,XY]. One cell line had two clones with chromosomal changes, each including an unbalanced translocation between chromosomes 8 and 14, plus one clone has a deletion of chromosome 7 at band F2 [40,XY,der(14)t(8;14)(C3;E5)/40,XY,del(7)(F2),der(14)t(8;14)(C3;E5)]. In the cell lines with structural abnormalities, five out of seven had extra chromosome 8 materials of various sizes ranging from the whole arm to a partial duplication of chromosome 8.

**Table 3:** Summary of structural anomalies in mouse ES cell lines

**Structural anomalies**	**Number of cell lines**
40,XY,der(2)t(2;8)(A1;A1)	1
40,XY,del(6)(B1C3)/40,XY	1
40,XY,t(6;12)(F3;D3)	1
40,XY,der(13)t(8;13)(A1;A1)	1
40,XY,der(14)t(8;14)(C3;E5)/40,XY,del(7)(F2),der(14)t(8;14)(C3;E5)	1
40,XY,der(17)t(8;17)(B2;E2)	2
**Total**	**7**

Six cell lines also had mixtures of both numerical and structural changes (Table 4). Among six cell lines, two cell lines had an extra Y chromosome, plus a derivative chromosome 1 due to an unbalanced translocation between chromosomes 1 and 8 [41,XY,+Y,der(1)t(1;8)(A1;D1)] (Figure 2C,D). One cell line had a translocation between chromosomes 4 and 14, plus an extra chromosome 6 [41,XY,t(4;14)(C6;E5),+6]. One cell line had three different clones, [41,XY,+3/40,XY,der(17)t(8;17)(B2;E2)/40,XY]. Another cell line also had three independent clones [40,XY,del(6)(B1C3)/41,XY,+4/40,XY]. The last cell line had two clones with a deletion of chromosome 10 and trisomy 8 [40,XY,del(10)(A4C1)/41,XY,+8,del(10)(A4C1)]. Four out of six cell lines had an extra chromosome 8 or a partially duplicated chromosome 8. The smallest partial duplications of chromosome 8 were from the band D1 to the terminus of chromosome 8, which found in the two cell lines with 41,XY,+Y,der(1)t(1;8)(A1;D1).

**Table 4:** Summary of both numerical and structural anomalies in mouse ES cell lines

**Structural and numerical anomalies**	**Number of cell lines**
41,XY,+Y,der(1)t(1;8)(A1;D1)	2
41,XY,t(4;14)(C6;E5),+6	1
41,XY,+3/40,XY,der(17)t(8;17)(B2;E2)/40,XY	1
40,XY,del(6)(B1C3)/41,XY,+4/40,XY	1
40,XY,del(10)(A4C1)/41,XY,+8,del(10)(A4C1)	1
**Total**	**6**

## Discussion

Analyses of mouse ES cell lines performed in our laboratory revealed a high rate of chromosomal abnormalities. Forty-four out of 97 ES cell lines (45%) showed abnormal karyotypes. Chromosomal abnormalities associated with chromosome 8, i.e., trisomy 8 or mosaic trisomy 8 or partial trisomy 8 due to an unbalanced translocation, accounted for 89% of all abnormalities (39 out of 44). Similar findings have been reported by other investigators [3,9,10]. ES cell clones with trisomy 8 have shown to have a selective growth advantage, and while they readily produce chimeras, they do not transmit the mutation to the germ line [3]. After trisomy 8, trisomy of chromosome 11 (6 out of 44) is the second most frequent abnormality in the karyotype analysis of ES cells, as also noted before [7,10].

The mechanism for these chromosomal abnormalities is not known, despite those frequencies. Generally, cells *in vitro* for numerous passages acquire chromosomal changes. The proportion of euploid cells starts to decrease abruptly after passage 15, and only 20 to 30% of cells remain euploid by passage 25 [5,11]. The efficiency of germ line transmission declines as the ES cell passage number increases in number, at least partially due to the presence of aneuploidy in the cell population [5,12].

On average, the ES cells used for this study had undergone at least fifteen to twenty passages. For this reason, we conclude that extended culture *in vitro* is at least one causative factor for the high frequency of chromosomal abnormalities. Although we were unable to identify all the mouse strains in our collection, we were able to positively identify 46 ES cell lines from four major mouse strains, AB2.2, CJ7, GSI-1, and J1. Among them, 15 cell lines were derived from the AB2.2 mouse strain, 24 from CJ7, 3 from GSI-1 and 4 from J1. Interestingly, we found that the cell line AB2.2 had chromosomal abnormalities with mosaic trisomy 8, [42,XY,+Y,+8/41,XY,+Y/40,XY]. Also, its sub-ES cell lines showed various karyotypic abnormalities.

Of the 11 sub-ES cell lines, two were normal, four were trisomy 8, one was mosaic trisomy 8, two were double trisomy with chromosomes 8 and 11 and one was derivative chromosome 14 resulting from an unbalanced translocation between chromosomes 8 and 14. The most interesting finding is that one of the sub-ES cell lines showed very complex numerical chromosome abnormalities, including the most common numerical abnormalities found in our study: 40,X,-Y,+8/40,X,-Y,+11/41,X,-Y,+6,+8/41,X,-Y,+6,+11/42,XY,+6,+8/42,XY,+6,+11/40, XY. We were not able to follow up on whether or not this cell line affected other ES cell lines.

## Conclusions

We have performed chromosomal analysis of 97 constructed ES cell lines. Chromosomal anomalies were seen in 44(45%) out of the specimens analyzed. We found trisomy 8 to be a common anomaly in the sub-ES cell lines identified from the original mouse strains. It is not uncommon that ES cells with trisomy 8 over-proliferate. This should serve as a warning for collaborative researchers attempting to maximize their limited resources by growing more and more ES cells before sharing their clones. Our findings indicate that before the injection of ES cells into blastocysts, karyotype analysis by conventional methods including FISH analysis is needed in order to ensure genome stability.

## Methods

All the experiments were performed on the established cell lines obtained from mice. No ethical issues is applied to this study.

### ES cell lines

Ninety-seven ES cell lines obtained from various research teams at the University of Oklahoma Health Sciences Center and the Oklahoma Medical Research Foundation between 2000 and 2006. The ES cell lines were of multiple origins, obtained either from commercial or academic sources, with different gene constructs. We were able to examine the AB2.2, CJ7, GSI-1 and J1 ES cell lines. Some cell lines originated from one single source and were expanded in different laboratories for at least another five to ten passages (1:3 or 1:4 splits) in culture under varying conditions. Thus, the cells used for this study had all undergone at least fifteen to twenty passages.

### Chromosome preparation and karyotype analysis

The standard procedures for harvesting, making slides and staining the cells were followed [8]. ES cells were arrested in metaphase by adding colcemid (final concentration of 0.02 μg/ml) to the culture medium for one hour. The cells were then washed in phosphate buffered saline (PBS). After trypsin treatment, detached cells were spun down. A hypotonic solution [0.075 M potassium chloride(KCl)] was added, and cells were incubated for 30 min at 37°C prior to fixation. Fixation with 3:1 methanol:glacial acetic acid was performed three times prior to spreading the cells on glass slides. Twenty cells were analyzed for each line and at least five cells were karyotyped with standard nomenclature [13].

### Fluorescent *in situ* hybridization (FISH)

The whole chromosome painting probes (WCP) for chromosomes 8, 10, 14 and 17 were purchased from a commercial source (Cambio, UK) for the ES cell lines with complex chromosomal abnormalities. FISH analysis was performed according to the manufacturer’s instructions.

## Abbreviations

ES: Embryonic stem; FISH: Fluorescence *in situ* hybridization; PBS: Phosphate buffered saline; KCl: Potassium chloride; WCP: Whole chromosome painting probes.

## Competing interests

The authors declare that they have no competing interests.

## Authors’ contributions

YK collected and/or assembly of data analysis and interpretation. JL carried data analysis and interpretation. LX provided of study material. JM participated in final approval of manuscript. SL conceived of the study concept and design, and participated in data analysis and interpretation, and final approval of manuscript. All authors read and approved the final manuscript.
